# Artificial Intelligence for Rapid Meta-Analysis: Case Study on Ocular Toxicity of Hydroxychloroquine

**DOI:** 10.2196/20007

**Published:** 2020-08-17

**Authors:** Matthew Michelson, Tiffany Chow, Neil A Martin, Mike Ross, Amelia Tee Qiao Ying, Steven Minton

**Affiliations:** 1 Evid Science El Segundo, CA United States; 2 InferLink El Segundo, CA United States; 3 Pacific Neuroscience Institute Providence St John’s Health Center Santa Monica, CA United States

**Keywords:** meta-analysis, rapid meta-analysis, artificial intelligence, drug, analysis, hydroxychloroquine, toxic, COVID-19, treatment, side effect, ocular, eye

## Abstract

**Background:**

Rapid access to evidence is crucial in times of an evolving clinical crisis. To that end, we propose a novel approach to answer clinical queries, termed rapid meta-analysis (RMA). Unlike traditional meta-analysis, RMA balances a quick time to production with reasonable data quality assurances, leveraging artificial intelligence (AI) to strike this balance.

**Objective:**

We aimed to evaluate whether RMA can generate meaningful clinical insights, but crucially, in a much faster processing time than traditional meta-analysis, using a relevant, real-world example.

**Methods:**

The development of our RMA approach was motivated by a currently relevant clinical question: is ocular toxicity and vision compromise a side effect of hydroxychloroquine therapy? At the time of designing this study, hydroxychloroquine was a leading candidate in the treatment of coronavirus disease (COVID-19). We then leveraged AI to pull and screen articles, automatically extract their results, review the studies, and analyze the data with standard statistical methods.

**Results:**

By combining AI with human analysis in our RMA, we generated a meaningful, clinical result in less than 30 minutes. The RMA identified 11 studies considering ocular toxicity as a side effect of hydroxychloroquine and estimated the incidence to be 3.4% (95% CI 1.11%-9.96%). The heterogeneity across individual study findings was high, which should be taken into account in interpretation of the result.

**Conclusions:**

We demonstrate that a novel approach to meta-analysis using AI can generate meaningful clinical insights in a much shorter time period than traditional meta-analysis.

## Introduction

The capacity of artificial intelligence (AI) to aggregate and process massive volumes of information is emerging as particularly crucial in the current moment, especially as the large amount of data available can be overwhelming for humans to evaluate [1]. AI technology can alleviate the burden of some of this overload by automatically processing the written text of medical papers, and converting the text into a more consumable, structured set of data that can be easily searched and analyzed. Essentially, AI turns all of the written articles into spreadsheets of results.

Further, although meta-analysis and systematic literature review are the gold standards for evidence [2], these analyses require significant time and effort to produce (often as long as 1 year [3]) and are therefore rarely updated [4,5].

Therefore, to produce this evidence in a more timely manner, we here propose the rapid meta-analysis (RMA). An RMA follows the same general framework methodology of a traditional meta-analysis, but leverages technology at each step, yielding a much faster time to production. Some data quality may be compromised due to the emphasis on fast time to production, but the ability to generate answers so quickly may warrant this tradeoff.

We were motivated to develop the RMA method based on a practical example of the current need for obtaining a rapid consensus on evidence from the literature. Hydroxychloroquine has been available since the 1950s [6] and has been used to treat malaria, lupus erythematosus, and rheumatoid arthritis. Most recently, hydroxychloroquine has been highlighted as a potential intervention to support patients with coronavirus disease (COVID-19). Although the efficacy outcomes of hydroxychloroquine are different in each clinical condition for which it is used, adverse events tend to be consistent. In this study, we used RMA to answer a specific clinical question regarding hydroxychloroquine and the degree to which ocular toxicity is a side effect. This is an important clinical question; however, we were not able to find a suitable aggregation of results.

The core innovation of an RMA is replacing as many of the steps of manual meta-analysis as possible with machine intelligence, as has been proposed previously [4,7]. Machines are not yet at the point where they can simply provide an answer to a posed question; therefore, RMA instead replaces as many manual steps as possible with machine assistance (or entirely AI). The goal is that each step could eventually be replaced with AI.

**Figure 1:**
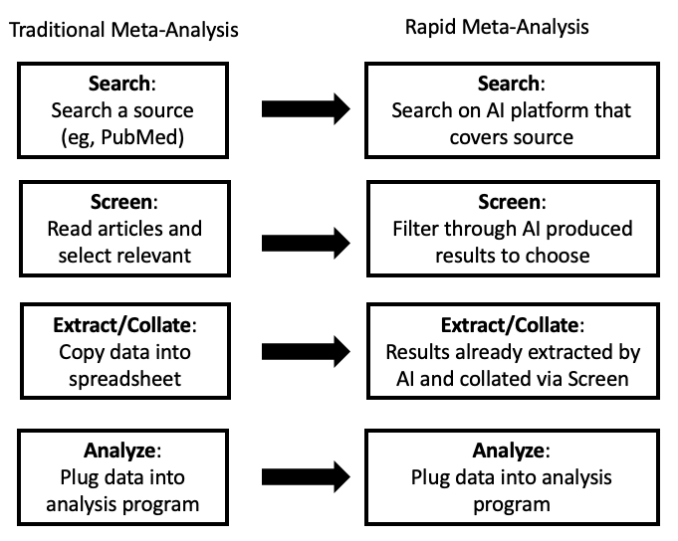
Traditional meta-analysis (simplified, left) vs rapid meta-analysis using artificial intelligence (AI) (right).

Figure 1 provides a schematic to make this idea more concrete. The left of the figure shows the standard steps (at a high level) for meta-analysis and the right side shows the equivalent steps with technology replacement.

For this RMA, we leveraged the Evid Science clinical outcomes database [8] for searching and screening (although any suitable AI system could provide a similar benefit). This database was built using the Evid Science AI, which is capable of turning written text of results into a “structured” representation (eg, a row in a database or spreadsheet).

**Figure 2:**
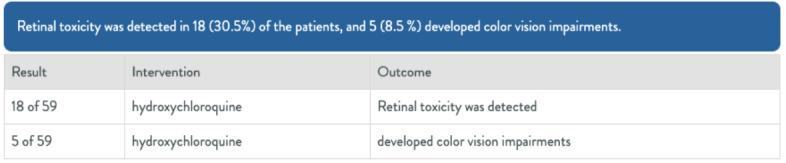
Example of artificial intelligence–generated results from article text.

Figure 2 shows a sentence from an article about toxicity detected for a set of patients, which has been parsed by the Evid Science AI. The AI was able to break this sentence down into fields (such as result, intervention, and outcome) automatically. In particular, it knows that 18 is the number of patients, and since that represents 30.5% of the patients, it must be 18 of 59. It also knows that 18 was associated with “Retinal toxicity being detected” in contrast to 5, which is associated with “color vision impairments.”

Previous AI-related approaches have attempted to identify sentences associated with Patient/Problem, Intervention, Comparison, Outcome (PICO) parameters from studies [9,10], surface more relevant articles for screening [11], and even study characteristics, including bias [12]. However, we were not able to find another AI that was purposely built to parse the full, numeric results from the text directly (eg, numbers and their associated fields), which are the inputs required for an advanced investigation such as a meta-analysis.

To train the AI to perform this task, researchers at Evid Science employed supervised machine learning. In this methodology, the researchers initially gave the system very explicit examples of the type of output they wanted; similar to the format shown in Figure 2, these comprised sets of sentences and the associated structured results.

The machine was then trained with a dataset of thousands of such examples from a wide variety of articles in the literature. The learning process enables the machine to produce these types of output for brand new sentences. To be clear, the articles chosen for training were selected from multiple disease topics and with various interventions, and were not only focused on hydroxychloroquine. As the system improves, it can even be taught to correct mistakes, rather than having to start with fresh examples each time, thereby limiting the effort involved in refining its learning.

## Methods

### Evid Science AI

The Evid Science AI is a deep-learning model, written in python, constructed from layers of transformers and bidirectional long-short term memory (bi-LSTM) units. Our model first encodes the inputs using the transformer language model (SciBERT [13]), which turns the words into a mathematical space where similar words are grouped together. These embedded inputs are then passed along through the bi-LSTM layers of the network, which traverse words in the text and labels them appropriately. We trained our algorithm on 24,614 labeled records.

### Model Performance

Recently, we performed a dual-annotator analysis of extraction accuracy. One hundred results were randomly selected from the database, each of which contains a result record (eg, numerator, denominator, percent, measurement value, unit, intervention/study group, outcome) and a sentence. Of note, a single sentence can be associated with multiple results; however, in randomizing, we chose one result to label for accuracy. Further, not all sentences have values for all fields. We then labeled the extracted fields (numerator, denominator, percent, measurement value, unit, intervention/study group, outcome) for accuracy. Our labels are provided as a spreadsheet in Multimedia Appendix 1.

The labels could be “perfect” (eg, a field was perfectly extracted); “near perfect” (eg, the field contained extra words or missed a few words, but was otherwise understandable, such as an outcome of “attained remission” contains the extra word “attained”); or “incorrect.” We also included “missing” as a means for estimating recall (true recall is hard to measure, given that we would require full labeling of all documents). The 100 sentences and labels are shown in Multimedia Appendix 1.

Specifically, for each field, we report the estimated recall, precision (which is accuracy), perfect precision (accuracy only considering perfect extractions), and F-measure (harmonic mean between recall and precision).

### Using the Model for RMA

From a practical perspective, in previous work, we demonstrated a similar process to RMA using the Evid Science AI to replicate the results from a systematic literature review [14]. Crucially, by leveraging AI, we produced the results in 6 days rather than the months it took to produce the original. In addition, given the time between the original publication and AI-assisted version, 22 new relevant results had been published. Of note, the current version of the AI used for this RMA is significantly more powerful than the version previously used for systematic literature review replication.

The Evid Science clinical outcomes database used in this RMA is the result of running the AI over the entirety of the publicly (freely) available medical literature (PubMed). The current database has nearly 70,000,000 “facts” associated with results from articles, which users can search and screen through.

**Figure 3:**
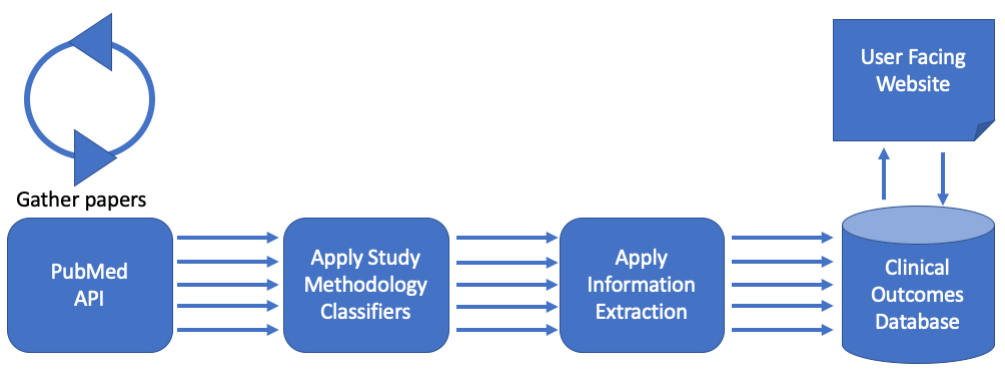
Evid Science artificial intelligence architecture. API: application programming interface.

The Evid Science platform has already indexed the entirety of PubMed, and each night, it pulls in the latest papers. The architecture for the system is shown in Figure 3. Starting at the left, articles come into the system via the PubMed application programming interface (API). Machine-learning classifiers are then applied to articles, determining the study type (eg, trial, observational study) and other methodology information. The extraction algorithm described above is then applied to the article. Each result from the extraction algorithm is then stored in our database and users can retrieve these results via searching and filtering via a web-based user interface. Final results can be exported (in CSV format) so that they can be analyzed in sophisticated statistical programs such as R.

Therefore, our RMA proceeds by searching and screening through this database, as described below. The search itself (Step 1) leverages PubMed APIs, and therefore returns equivalent articles to PubMed. That is, any search on our platform is passed to the PubMed API, and the returned articles are then matched against what our AI has extracted. Therefore, the initial search results are equivalent.

Screening is then simplified, since the AI has processed the text into structured records that can be filtered and screened efficiently. For instance, we can simply filter results associated with “toxicity” in the outcome (or other outcomes of interest). This is more efficient than manually reading each returned abstract, since one only screens articles in the filtered set.

After searching and screening, a user obtains the final dataset for analysis (which the AI helped to produce via extraction). One can perform many analyses directly within the Evid Science web-based tool or export data to Excel and then analyze it with other programs (as we have done).

## Results

### RMA Process for the Association of Eye Issues With Hydroxychloroquine Use

We initially performed a search for hydroxychloroquine on the Evid Science platform, and filtered down to results in which the outcome was major vision impairments (eg, “maculopathy,” “blind,” “toxicity”). In this study, we focused solely on PubMed abstracts, since they are freely available. This yielded 22 candidate articles from a possible set of 5010 articles related to hydroxychloroquine, 1352 of which were identified as primary studies (eg, clinical trial or observational study) by our AI and were therefore included as possible articles to process results from.

After screening, we were left with 11 papers for our RMA; the other 11 excluded articles were published before 2000 or focused on the diagnosis of ocular issues. The search took less than 1 minute and the screening took 22 minutes. Most of the work involved selecting the papers and lightly cleaning the results to make the table of results easier to read. Two results required “more significant” human intervention: one result was reported as having “all cases” of documented blindness attributed to causes other than hydroxychloroquine, and we therefore needed to invert this result to be 0 cases attributed to hydroxychloroquine; the other result had a misattributed denominator, which was manually fixed. All other changes involved removing single words, which was required very infrequently in concordance with the accuracy results shown in Table 1.

The results from the search and screening processes are shown in Table 2, which served as the input for our meta-analysis computation. Although we did not conduct the equivalent screening manually, in previous work, we were able to use our AI to match a published systematic literature review on inflammatory bowel disease [14], and therefore have already demonstrated that we can generate the equivalent screening using our tool to that obtained with a manual process.

**Table 1:** Extraction results based on 100 randomly selected results, dual-screened.

Result extracted	Recall	Precision	Perfect Precision	F-measure	F-measure (Perfect)
Numerator (N=42)	92.86%	95.12%	95.12%	93.98%	93.98%
Denominator (N=23)	91.30%	91.30%	91.30%	91.30%	91.30%
Percent (N=40)	90.00%	94.74%	92.11%	92.31%	91.04%
Continuous Value (N=29)	89.66%	92.86%	92.86%	91.23%	91.23%
Continuous Unit (N=24)	83.33%	95.24%	90.48%	88.89%	86.76%
Intervention (N=85)	84.71%	94.74%	86.84%	89.44%	85.76%
Outcome (N=100)	95.00%	97.94%	79.38%	96.45%	86.49%

**Table 2:** Results from included papers.

Events (n)	Patients (n)	Intervention	Outcome	Reference
2	400	patients who were treated with recommended dosages of the drug for a mean of 8.7 years	incidence of hydroxychloroquine-related retinopathy	Mavrikakis et al [15]
0	526	hydroxychloroquine	retinal toxicity was noted during the first 6 years of treatment	Mavrikakis et al [15]
46	845	chloroquine, hydroxychloroquine, or both	ophthalmological alterations, confirmed by the ophthalmological examination	Spinelli et al [16]
3	12	800 mg/day hydroxychloroquine	developed retinal toxicity with scotomas in the Amsler grid and Humphrey 10-2 automated perimetry, as well as abnormal multifocal electroretinography	Navajas et al [17]
0	11	long-term hydroxychloroquine	documented blindness, in all cases attributed to a cause other than hydroxychloroquine-related ocular toxicity	Singh et al [18]
35	678	hydroxychloroquine	had hydroxychloroquine toxicity	Chiu et al [19]
1	121	hydroxychloroquine	prevalence of toxic retinopathy	Cabral et al [20]
18	59	hydroxychloroquine	retinal toxicity was detected	Espandar et al [21]
9	778	antimalarial drugs	suffered definite presence of antimalarial retinopathy	Jover et al [22]
11	36	hydroxychloroquine	had abnormal response densities in one or both eyes	Maturi et al [23]
3	26	hydroxychloroquine	results from electrophysiological and clinical evaluation, toxicity (bull’s eye maculopathy)	Tzekov et al [24]
4	93	chloroquine and hydroxychloroquine therapy	developed typical bull’s eye maculopathy	Neubauer et al [25]

### RMA Outcome

We then performed a meta-analysis of the results from the screened out articles using a generalized linear mixed model (in R), as these are binary occurrences of having an eye issue. We chose a random-effects model for the analysis, demonstrating a result of 3.4 events of eye issues per 100 observations (95% CI 1.11-9.96). The code for this analysis was already written; therefore, plugging in the data (Table 2) and running it took roughly 2 minutes, including exporting the data to Excel, renaming and selecting columns to conform to R code input, and running the code.

**Figure 4:**
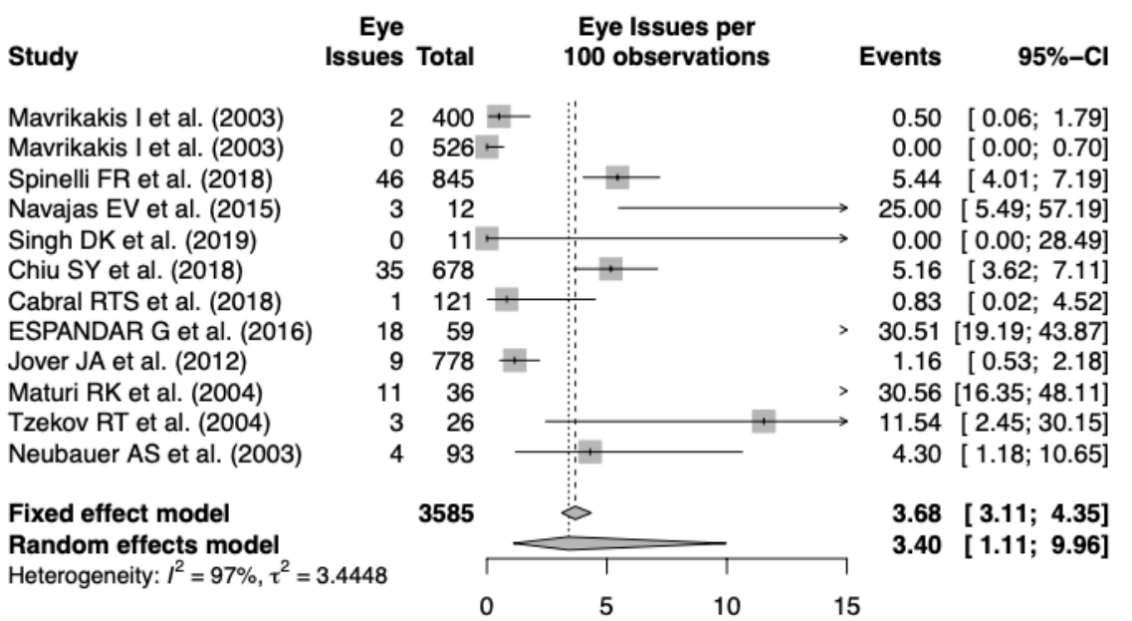
Forest plot for our rapid meta-analysis.

**Figure 5:**
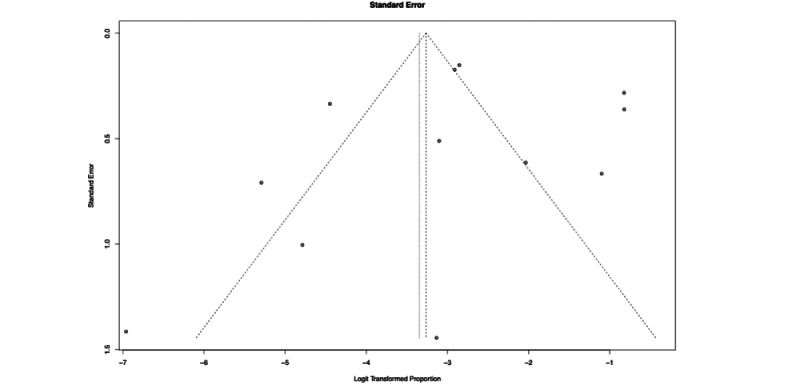
Funnel plot for our rapid meta-analysisis.

**Figure 6:**
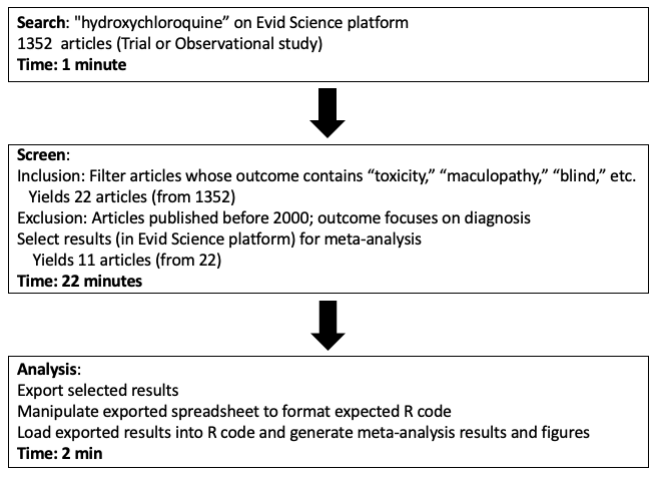
Overview of the rapid meta-analysis for ocular toxicity associated with hydroxychloroquine.

The forest plot of the meta-analysis is shown in Figure 4. Clearly, there was heterogeneity (I^2^=97%) among studies; therefore, these results warrant deeper inspection and cautious interpretation. The funnel plots of the results are shown in Figure 5. Each step of our RMA, its output, and its timing are shown in Figure 6, showing that altogether the RMA process took roughly 30 minutes to complete.

## Discussion

### Principal Findings

For the RMA, we leveraged the Evid Science clinical outcomes database to find relevant studies, screened the database for studies focused on hydroxychloroquine and vision issues, and then performed the meta-analysis computation. Most crucially, the entire process from the search to analysis took less than 30 minutes. Based on results from 11 studies (N=3585), we could expect to see major eye issues 3.4% (95% CI: 1.11%-9.96%) of the time when using hydroxychloroquine. We note the high heterogeneity across the studies (I^2^=97%), requiring caution when interpreting these results. Notably, an RMA such as the present analysis is meant to raise awareness, and should not be treated as a full systematic literature review or meta-analysis that should strictly guide treatment. The data and results presented herein are current as of April 11, 2020.

The screening of articles step is one of the key areas of time savings in RMA. Although we described the various accuracy metrics in Table 1, the AI is also able to surface study characteristics that can be helpful in screening for meta-analysis as well. For example, Table 3 shows various study characteristics for a few of the chosen articles, including time period (retrospective or prospective), cohort focus (groups designated based on different drugs or conditions), study type (trial or observational), and finally methodology sentences. These characteristics are all generated by the machine, except for the methodology sentences, which are surfaced from the text automatically (rather than applied as “tags” to an article). In our RMA, we chose to include as many data points as possible, which perhaps led to our high level of heterogeneity (although population size differences clearly influence heterogeneity as well), but we could have been more specific, focusing on certain study characteristics using the values supplied by the AI. For instance, the methodology sentences include geographic locations, or we could have focused solely on papers that group patients by drug, rather than condition. For a complete list of characteristics for all articles, refer to Multimedia Appendix 2.

**Table 3:** Study characteristics, pulled via artificial intelligence (see Multimedia Appendix 2 for the characteristics of all papers).

Methodology Sentences	Time period	Cohort focus	Study type	Reference
The incidence of irreversible retinal toxicity in patients treated with hydroxychloroquine: a reappraisal. To define the risk of hydroxychloroquine (HCQ)-related retinal toxicity in patients with rheumatoid arthritis (rheumatoid arthritis) and systemic lupus erythematosus (systemic lupus erythematosus) who are receiving recommended dosages of the drug (< or =6.5 mg/kg/day). Prospective cohort study, from 1985 to 2000. Greek patients with rheumatoid arthritis (n=335) and systemic lupus erythematosus (n=191) treated with hydroxychloroquine, 400 of whom had completed at least 6 years of treatment.	Prospective	Drug Therapy	Observational	Mavrikakis et al [15]
Treating lupus patients with antimalarials: analysis of safety profile in a single-center cohort. This longitudinal retrospective study aims at describing the safety profile and the reasons for discontinuation of antimalarials in patients with systemic lupus erythematosus (systemic lupus erythematosus) and discoid lupus erythematosus (discoid lupus erythematosus), focusing on ocular toxicity. We analyzed the clinical data of 845 systemic lupus erythematosus and discoid lupus erythematosus patients; 59% of them were taking antimalarials: 1.4% chloroquine (chloroquine), 88.5% hydroxychloroquine (hydroxychloroquine) and 10.1% both.	Retrospective	Drug Therapy	Observational	Spinelli et al [16]
Retinal toxicity of high-dose hydroxychloroquine in patients with chronic graft-versus-host disease. To evaluate retinal toxicity in patients treated with high-dose hydroxychloroquine (hydroxychloroquine) (Plaquenil, Sanofi Pharmaceuticals) for chronic graft-versus-host disease (graft versus host disease). Twelve patients with chronic graft versus host disease treated with 800 mg/day hydroxychloroquine between June 2005 and December 2010.	Retrospective	Drug Therapy	Observational	Navajas et al [17]

### Limitations

Of course, there are limitations to our approach as well. One important aspect to note is that although RMA can very rapidly produce answers to clinical questions, nothing (yet) replaces human ingenuity and creativity (and most importantly, common sense). A major limitation of RMA currently is that the AI is not sophisticated enough to present more than data and mathematical results; that is, it cannot make meaningful interpretations.

In this case, for instance, the result is indicative (3.4 events per 100 observations), but the confidence interval is wide and the I^2^ is high. Therefore, the result of our RMA warrants cautious interpretation. A machine cannot produce this nuance summary but can only provide the data and results for a person to then interpret.

Another limitation is that models may make mistakes. Of course, human beings make mistakes as well, but model mistakes can be counterintuitive. For instance, in this study, one of the results extracted was that all cases had documented blindness associated with something other than hydroxychloroquine. This implies zero cases for hydroxychloroquine, but the machine did not pick up on this. It is obvious to us as humans that the inverse result is what we want, but that is a common sense observation. Therefore, there is a tradeoff between assuming there will be some mistakes and the rapid nature of RMA. We note that there are often mistakes in human analysis as well.

A final limitation is acceptability. We introduce RMA as a means to more rapidly produce evidence that can be helpful in clinical decision making. However, without trust in and the adoption of AI-assisted evidence, such results might exist in a vacuum. If that is the case, clinical practice will not benefit from the advancement. Therefore, this limitation necessitates that, to be useful, current AI-generated evidence must be accepted in some manner. We hope that our transparency in this article (presenting results and data) helps to bring some change in this regard.

### Conclusion

In this article, we have presented a new framework for answering clinical questions when time is at a premium and can be traded off for data quality. We call this approach RMA, and we demonstrated its utility in answering a clinical question about ocular toxicity associated with hydroxychloroquine as a proposed treatment for COVID-19. By leveraging RMA, in roughly 30 minutes we were able to discern a potential association with an incidence of 3.4 events per 100 observations (95% CI 1.11-9.96). Although the results raise further questions that need to be considered (eg, regarding the high heterogeneity), they nevertheless raise attention to a relevant clinical issue with the drug hydroxychloroquine. Importantly, the whole assessment was completed in less than 30 minutes, representing huge time savings compared to the months it takes for traditional meta-analysis conducted by hand.

## Appendix

Multimedia Appendix 1 Labels from dual-annotator labeling of extractions.

Multimedia Appendix 2 The full study characteristics for Table 3.

## Abbreviations

AI: artificial intelligence
API: application programming interface
bi-LSTM: bidirectional long-short term memory
COVID-19: coronavirus disease
RMA: rapid meta-analysis
